# Effect of preharvest biofilm application regimes on cracking and fruit quality traits in ‘0900 Ziraat’ sweet cherry cultivar

**DOI:** 10.1186/s12870-024-05224-z

**Published:** 2024-06-18

**Authors:** Burhan Ozturk, Husrev Akkaya, Erdal Aglar, Onur Saracoglu

**Affiliations:** 1 Faculty of Agriculture, Department of Horticulture, Ordu University, Ordu, Türkiye; 2 Faculty of Agriculture, Department of Horticulture, Van Yüzüncü Yıl University, Van, Türkiye; 3 Faculty of Agriculture, Department of Horticulture, Tokat Gaziosmanpaşa University, Tokat, Türkiye

**Keywords:** Antioxidant activity, Firmness, Respiration rate, Sweet cherry, Total phenolics, Total flavonoids

## Abstract

**Background:**

Fruit cracking impacts the quality of sweet cherry, significantly affecting its marketability due to increased susceptibility to injury, aesthetic flaws, and susceptibility to pathogens. The effect of 1% biofilm (Parka™) application regimes on fruit cracking and other quality parameters in the ‘0900 Ziraat’ cherry cultivar was investigated in this study. Fruit sprayed with water were served as control (U1). Fruit treated only once with biofilm three, two and one week before the commercial harvest were considered as U2, U3 and U4, respectively. Fruit treated with biofilm three, two, and one week before harvest were considered as U5; three and two week before harvest as U6; two and one week before harvest as U7; and fruit treated three and one week before harvest as U8.

**Results:**

In both measurement periods, the lower cracking index was obtained in biofilm-treated sweet cherry fruit. However, the firmness of biofilm-treated fruit was higher than that of the control fruit. The lowest respiration rate was observed in U7, while the highest weight was recorded in U4 and U5 than the control. The biofilm application decreased fruit coloration. The biofilm application also increased the soluble solids content of the fruit. The U2, U3 and U4 applications at harvest showed higher titratable acidity than the control. In both measurement periods, the vitamin C content of the U2, U5, U6, U7 and U8 applications was found to be higher than that of the control. The total monomeric anthocyanin of the U3 and U8 applications was higher than that of the control. Furthermore, the antioxidant activity of the U2, U3 and U5 in the DPPH, and the U7 and U8 in FRAP were measured higher thanthat of the control.

**Conclusions:**

The application of biofilms has the potential to mitigate fruit cracking, prolong postharvest life of sweet cherries, and enhance fruit firmness.

## Background

With its diverse climate and soil characteristics, Türkiye is the global leader in sweet cherry production. Its exceptional quality is a key factor in the country’s ability to cater to consumer preferences. Nevertheless, the brief duration of the sweet cherry harvest and the fragility of its texture restrict its market availability to a few weeks [1]. The rapid softening, susceptibility to fungal infections, and vulnerability to mechanical damage result in a loss of fruit quality of up to 15% [2]. Efforts to minimize quality losses, prolong the postharvest shelf life of seet cherries, and reduce transportation-related damage involve preharvest applications of coatings and plant growth regulators which aim to enhance fruit quality [3, 4] and postharvest interventions to slow fruit ripening [5]. The quality of sweet cherry is significantly influenced by a number of factors, including environmental, morphological, physiological, and genetic factors [6]. The primary cause of fruit cracking is increased turgor pressure within the fruit due to its water potential. Factors such as preharvest rain and soil water absorption have been shown to significantly increase turgor pressure [7]. Furthermore, the cultural practices and environmental conditions affecting fruit development, fruit characteristics, and skin anatomy and firmness influence cracking in sweet cherry, plum, apricot, citrus, litchi, pomegranate, apple, banana, grape, avocado, persimmon and peach fruits [8]. Peel mechanical properties, which are influenced by factors such as calcium content, pectin values, cell wall structure, and the amount and volume of the intercellular spaces [9], play a significant role in resistance against fruit cracking. A number of morphological properties, including cuticle thickness and physical properties, the number of hypodermal layers [10], fruit shape, and fruit size [11] also affect fruit cracking susceptibility in fruit species such as tomato [12], sweet cherry [13], apple [14], and nectarine [15].

A variety of strategies were employed in preharvest applications including the use of rain cover protection [16], gibberellic acid [16], methyl jasmonate [17], seaweed extracts [18], calcium [18], glycine betaine [19] and putrescine [20] with the objective of preventing fruit cracking in sweet cherry. The majority of studies addressing fruit cracking have focused on reducing fruit water potential and increasing fruit calcium content [21]. The objective of the calcium-based chemical applications is reduce water uptake through the fruit cuticle prior to rainfall [22]. Previous studies have demonstrated the efficacy of calcium applications in mitigating rain-induced cracking in sweet cherry [23] by strengthening cell walls [24]. Postharvest calcium applications in sweet cherries have been shown to reduce disorders such as fruit rot and spot formation, while maintaining fruit quality [25]. Biofilm formulations have shown efficacy in reducing water uptake, enhancing skin elasticity, and decreasing fruit cracking rates in sweet cherry [26]. However, the majority of existing studies have employed single-spray applications at a 1% concentration, with a paucity of research exploring into application regimens.

Therefore, the main study question was: what is the effect of application regimens on fruit cracking and quality traits of sweet cherry? This research hypothesized that biofilm application regimens will have a significant positive effect on delaying or reducing of fruit cracking in sweet cherries. This study aims to address this gap by investigating the effect of biofilm application regimes on fruit cracking and other fruit quality characteristics in the ‘0900 Ziraat’ cherry cultivar.

## Materials and methods

### Plant material

Nine-year-old sweet cherry (*Prunus avium* L. cv. ‘0900 Ziraat’) trees grafted on MaxMa 14 rootstock, located in an orchard at the Tokat Gaziosmanpaşa University Agricultural Application and Research Center (40° 20’ 02.19” N latitude, 36° 28’ 30.11” E longitude, 623 m altitude), were used as the plant material. The trees were planted in an east-west direction, with a spacing of 5 m between rows and 3 m between trees utilizing the Vogel Central Leader system. The orchard was subjected to regular fertilization, irrigation, and weed control.

### Experimental design

The study employed a randomized block design with three blocks. For each block, one tree was selected for each application of 1% Parka (5% cellulose, 7.5% stearic acid, and 1% calcium; Cultiva, USA), with uniform growth vigor and product load. A buffer tree was positioned between each application tree to prevent any potential interference with the application process. Spraying commenced three weeks prior to harvest at which point the fruit had turned yellow-straw color. Two subsequent applications were performed at one-week intervals. A 0.05% concentration of ‘Sylgard 309’ (Dow Corning, Canada) was added to the spray solution in order to enhance adhesion. The solution was applied to the entire tree canopy by means of spraying. The control trees were treated a solution comprising water and surfactant only. The spraying was conducted on mornings devoid of precipitation and wind, utilizing a low-pressure back pump. The application regimes were implemented in accordance with the specifications outlined in Table 1.

**Table 1:** Pre-harvest biofilm (1%) application regimes in sweet cheery

Application	Weeks before anticipated commercial harvest		
	3	2	1
U1 (control)	–	–	–
U2	1%	–	–
U3	–	1%	–
U4	–	–	1%
U5	1%	1%	1%
U6	1%	1%	–
U7	–	1%	1%
U8	1%		1%

In the commercial harvest (22 June 2019), one hundred fruit were hand-harvested in each of the trial trees. In addition, one hundred fruit were again collected in the trial trees one week after the commercial harvest (29 June 2019) to simulate the efficacy of biofilm in reducing fruit cracking when harvesting is delayed. The harvested fruit were transported in a refrigerated vehicle to the Post-Harvest Physiology Laboratory of the Department of Horticulture, Faculty of Agriculture, Tokat Gaziosmanpaşa University where pomological and biochemical analyses were conducted.

### Physiological and physico-chemical parameters

The fruit weight (fifty fruit in each rep) was determined by weighing them on a digital scale (Radwag PS/C/1, Poland) with a precision of 0.01 g. The fruit sizes (fifty fruit in each rep) were determined by a digital caliper (Mitutoyo, Japan) with a precision of 0.01 mm and expressed in mm. The color of fruit (ten fruit in each rep) was quantified using the CIE L*, a*, and b* color space with measurements taken on two opposite poles of the equatorial part using a colorimeter (Minolta, model CR-400, Tokyo, Japan). Fruit firmness (ten fruit in each rep) was assessed on opposite cheeks of the equatorial part of the fruit using a digital penetrometer (Agrosta 100 field, Agrotechnologie, France) with the 10-point tip of the device perpendicularly. Ten fruit in each replication were sealed in 2 L jars at 20 ± 1 °C and 90% relative humidity for 1 h (h). The amount of CO_2_ released during this period was quantified using a digital CO_2_ sensor (Vernier Software, Oregon, USA), expressed as nmol CO_2_ kg^–1^ s^–1^ [16]. The juice obtained from ten fruit in each replication after blending and passing through cheesecloth was employed to ascertain the soluble solids content (SSC) utilizing a digital refractometer (PAL-1, Atago, USA). Titratable acidity was determined by titrating a juice sample (10 mL) diluted in 10 mL distilled water with 0.1 mol L^–1^ (N) sodium hydroxide (NaOH) until the pH reached 8.1 The amount of NaOH consumed in the titration was expressed as grams of malic acid per 100 mL.

### Cracking index

A total 50 fruit were selected from each application in each block and subjected to a water immersion test in a container filled with 5 L water (20 ± 1 °C). The number of cracked fruit was recorded at 2, 4, and 6 h intervals, with cracked fruit removed from the water at each time interval. The cracking index was calculated using the Eq. (1), where a, b, and c represent the number of cracked fruit after 2, 4, and 6 h, respectively [27].

1 $${\rm{Cracking}}\,{\rm{index}}\, = \,\left( {5{\rm{a }} + {\rm{ }}3{\rm{b }} + {\rm{ c}}} \right)\, \times \,100/250$$

### Vitamin C

A reflectoquant plus device (Merck RQflex plus 10, Türkiye) was employed to quantify vitamin C (ten fruit in each rep). Initially, 0.5 mL of the previously obtained extract was combined with 4.5 mL of 0.5% oxalic acid. The solution was then immersed in an ascorbic acid test kit (Catalog no: 116,981, Merck, Germany) for a period of 2 s (s), followed by an 8 s wait for oxidization to occur outside the solution. The reflectoquant device was inserted into the test adapter in order to obtain readings, which were expressed as milligrams (mg) per hundred grams (mg 100 g^–1^) [16]. The vitamin C was duplicated three times for each replicate.

### Total phenolics, total flavonoids and total anthocyanin content

For the determination of total phenolics, total flavonoids and total anthocyanin content including antioxidant analysis, the stones of ten randomly harvested fruit from each replicate were separated from the pulp. Subsequently, the pulp was then homogenised using a blender, and approximately 50 g of pulp was kept in falcon tubes at -80 °C in a deep freezer until analysis. The total phenolics, flavonoids and anthocyanin contents including antioxidant analysis were duplicated three times for each replicate. The main solvent used for extraction was methanol.

The Folin-Ciocalteu’s chemical method, as described in Ozturk et al. [28] was employed to determine the total phenolics. A 600 µL extract was combined with 4.0 mL distilled water, followed by the addition of 100 µL Folin-Ciocalteu’s reagent and 300 µL of 2% sodium carbonate (Na_2_CO_3_). Following a two h incubation period at room temperature, the greenish solution was read at 760 nm using a UV-vis spectrophotometer (Shimadzu, Japan). The results were expressed as mg gallic acid (GAE) per 100 g fresh weight (fw).

A modified method of Zhishen et al. [29] was employed to determine total flavonoids. A 600 µL extract was combined with 3.7 mL methanol, followed by the addition of 100 µL of 10% aluminum nitrate [Al(NO_3_)_3_] and 0.1 M ammonium acetate (NH_4_CH_3_CO_2_). Following 40 min (min) incubation period in the absence of light at room temperature, the absorbance was read at 415 nm using a UV-vis spectrophotometer, with the results expressed as mg quercetin equivalent (QE) per 100 g fw.

The pH difference method, as described by Giusti et al. [30], was employed to quantify total monomeric anthocyanin (TMA) in fruit extracts prepared in pH 1.0 and 4.5 buffers. Absorbance was measured at 533 and 700 nm using a UV-vis spectrophotometer. Total anthocyanin amount (molar extinction coefficient of 29,600 cyanidin-3-glucoside) absorbances [(A520–A700) pH 1.0 – (A520–A700) pH 4.5] were expressed as micrograms (µg) cyanidin-3-glycoside per gram of fresh weight.

### Total antioxidant activity

The Blois [31] method with modifications [28] was used to measure DPPH free radical scavenging activity. A 500 µL extract was mixed with 2.5 mL ethanol and 0.5 mL of 0.1 mM ethanolic solution of DPPH. The solution was vortexed for 1 min and placed in a dark environment for 30 min at room temperature. The absorbance of the solution was read at 517 nm using a UV-vis spectrophotometer and the results were expressed as millimole (mmol) Trolox equivalent (TE) per 100 g fw.

The FRAP analysis involved preparing a 0.2 M phosphate buffer (pH 6.7) and adding 1.15 mL to 100 µL extract with 1.25 mL of 1% potassium ferricyanide [K_3_Fe(CN)_6_]. The reaction mixture was incubated at 50 °C for 20 min and cooled to room temperature before adding 1.25 mL of 10% trichloroacetic acid (TCA) and 0.25 mL of 0.1% iron chloride (FeCl_3_) and vortexing for 1 min. The absorbance of the solution was read at 700 nm using a UV-vis spectrophotometer. The results were expressed as millimole (mmol) Trolox equivalent (TE) per 100 g fw.

### Statistical analysis

Normality of the data was tested using the Kolmogorov-Smirnov test and homogeneity of variance using the Levene test. Descriptive statistics were assessed using analysis of variance. After analysis of variance, Tukey’s multiple comparison test was used to determine the level of significance between applications. Statistical analyses were performed using the SAS package program (SAS 9.1 version, USA), with a significance level of α = 5%.

## Results

### Fruit weight, fruit sizes and fruit color

The significant differences in fruit weight were observed between the different applications at the commercial harvest date and one week later. The U4 application had the highest fruit weights (7.68 and 8.04 g, respectively), while the U6 application had the lowest (5.64 and 5.88 g, respectively). In addition to, fruit weights measured in U5 were significantly higher than the other applications. While fruit weight did not change significantly with delay harvest in most applications, the U4 application showed variation with harvest delay (Table 2).

**Table 2:** Effect of biofilm application on fruit size of ‘0900 Ziraat’ sweet cherry cultivar

Application		Fruit size							
	Fruit weight (g)				Fruit width (mm)			Fruit length (mm)	
	Harvest		Harvest + 7days		Harvest	Harvest + 7days		Harvest	Harvest + 7days
U1	6.35 c-A		6.72 c-A		23.24 b-A	23.41 c-A		21.59 c-B	22.32 c-A
U2	6.27 c-A		6.58 c-A		22.42 c-B	23.21 c-A		21.53 c-B	22.41 c-A
U3	6.02 d-A		6.22 d-A		22.44 c-A	22.73 d-A		22.21 b-A	22.35 c-A
U4	7.68 a-B		8.04 a-A		24.05 a-B	24.66 a-A		23.32 a-B	24.08 a-A
U5	7.18 b-A		7.36 b-A		23.46 b-B	24.02 b-A		22.34 b-B	23.50 b-A
U6	5.64 e-A		5.88 e-A		22.07 d-A	22.12 e-A		21.56 c-A	21.75 d-A
U7	6.00 d-A		6.20 d-A		22.32 c-A	22.72 d-A		21.49 c-B	22.37 c-A
U8	6.38 c-A		6.59 c-A		22.28 c-B	22.84 d-A		21.48 c-B	22.54 c-A

Means in columns with the same lowercase letters do not significantly differ according to Tukey’s test at *P* < 0.05. Means in rows with the same uppercase letters do not significantly differ according to Tukey’s test at *P* < 0.05

A significant differences in fruit width were observed between the different applications at the commercial harvest date and one week later. The U4 application exhibited the greatest fruit width at the commercial harvest (24.05 mm) and one week later (24.66 mm), while the U6 application exhibited the smallest fruit widths (22.07 and 22.12 mm, respectively). The U4 application had exhibited the greatest fruit length (23.32 mm), followed by U3 (22.21 mm) and U5 (22.34 mm). The U1, U2, U4, U5, U7, and U8 applications had significantly higher values at harvest + 7days than at the commercial harvest (Table 2).

The biofilm applications had a significant impact on the L*, a*, and b* color values. At the commercial harvest, the L* values of the U1 and U3 applications were found to be similar, with the former exhibiting significantly higher values. One week later, the L* value of the U1 application was found to be significantly higher than that of the other applications, while the L* value of the U8 application was found to be significantly lower (Table 3).

**Table 3:** Effect of biofilm application on fruit color of ‘0900 Ziraat’ sweet cherry cultivar

Application	Color values							
	L*			a*			b*	
	Harvest	Harvest + 7days		Harvest	Harvest + 7days		Harvest	Harvest + 7days
U1	37.03 a-A	33.65 a-B		35.36 a-A	29.95 a-B		16.82 a-A	12.20 a-B
U2	33.26 b-A	31.97 b-B		32.70 b-A	26.84 b-B		15.99 a-A	8.92 b-B
U3	36.64 a-A	31.85 b-B		35.60 a-A	26.51 b-B		16.74 a-A	9.19 b-B
U4	36.83 a-A	31.61 b-B		34.94 a-A	26.48 b-B		16.86 a-A	8.92 b-B
U5	33.01 b-A	31.43 b-B		30.41 c-A	26.17 b-B		12.02 b-A	8.52 b-B
U6	32.95 b-A	31.19 b-B		30.26 c-A	23.90 c-B		11.82 b-A	8.51 b-B
U7	32.55 b-A	31.01 b-B		28.74 d-A	24.11 c-B		9.97 c-A	8.68 b-A
U8	32.77 b-A	29.98 c-B		30.80 c-A	24.54 c-B		11.48 b-A	7.84 b-B

Means in columns with the same lowercase letters do not significantly differ according to Tukey’s test at *P* < 0.05. Means in rows with the same uppercase letters do not significantly differ according to Tukey’s test at *P* < 0.05

At the point of commercial harvest, the U3 and U4 applications exhibited a* values that were comparable to those of the control (U1), and were significantly higher than those of the other applications. The a* values of the U5, U6, and U8 applications had similar, significantly lower than the U2 application and significantly higher than the U7 application. One week later, all biofilm applications exhibited lower a* values than the control. However, the U6, U7, and U8 applications had similar a* values, which were significantly lower than those observed for the other applications. All applications exhibited significantly higher a* values at the time of commercial harvest than one week later (Table 3).

At the commercial harvest, the U5, U6, U7, and U8 applications had significantly lower b* values than the control (U1). The U7 application had significantly lower b* values than the others. One week later, the biofilm applications had similar b* values, which were significantly lower than those of the control (Table 3).

### Respiration rate, fruit firmness, and fruit cracking

At the time of the commercial harvest and one week later (harvest + 7th day), the U7 application had a significantly lower respiration rate than the control and other biofilm applications. At the commercial harvest, the U4, U5, and U6 applications exhibited significantly lower respiration rates than the control (U1). However, one week later, the U2 and U8 applications exhibited significantly higher respiration rates than the control (Table 4).

**Table 4:** Effect of biofilm application on respiration ratio, fruit firmness, and fruit cracking index of ‘0900 Ziraat’ sweet cherry cultivar

Application	Fruit characteristics							
	Respiration ratio (nmol kg s^–1^)			Fruit firmness (*N*)			Cracking index	
	Harvest	Harvest + 7days		Harvest	Harvest + 7days		Harvest	Harvest + 7days
U1	19.50 a-A	18.33 b-B		47.18 b-A	45.30 b-B		8.85 a-A	6.24 a-A
U2	20.02 a-A	19.86 a-A		58.12 a-A	52.70 a-B		2.80 b-A	1.05 c-B
U3	20.70 a-A	18.47 b-B		57.94 a-A	53.29 a-B		0.65 d-B	1.20 c-A
U4	18.59 b-A	18.21 b-A		60.07 a-A	53.25 a-B		0.50 d-B	0.90 c-A
U5	18.25 b-A	18.73 b-A		61.36 a-A	53.54 a-B		2.84 b-A	2.67 b-A
U6	18.99 b-A	18.47 b-A		62.33 a-A	52.89 a-B		0.50 d-B	0.90 c-A
U7	16.62 c-A	15.07 c-B		59.67 a-A	53.67 a-B		1.32 c-B	2.45 b-A
U8	20.20 a-A	19.90 a-A		60.46 a-A	54.82 a-B		1.45 c-B	2.45 b-A

Means in columns with the same lowercase letters do not significantly differ according to Tukey’s test at *P* < 0.05. Means in rows with the same uppercase letters do not significantly differ according to Tukey’s test at *P* < 0.05

At the commercial harvest and one week later, the biofilm applications resulted in significantly firmer fruit than the control. In addition, at the commercial harvest, the U6 application exhibited significantly firmer fruit (62.33 N) than the other biofilm applications. One week later, the U8 application had significantly higher values (54.82 N) than the other biofilm applications (Table 4).

The application of biofilms resulted in a notable reduction in the cracking index in both measurement periods. The lowest cracking index was observed in the U3, U4, and U6 applications at the commercial harvest, while the lowest index was observed in the U2, U3, U4, and U6 applications had the lowest one week later. The U3, U4, U6, and U7 applications exhibited significantly lower cracking index values at the commercial harvest compared to one week later. At the time of harvest plus seven days, the U2 application exhibited a significantly lower cracking index than U1, U5, U7 and U8. The control (U1) and U5 applications had similar levels of fruit cracking in both measurement periods (Table 4).

### Soluble solids content, titratable acidity, and vitamin C

At the commercial harvest, the U2, U4, U5, U6, U7, and U8 applications had significantly higher SSC than the control (U1) fruit. One week later, the U3 application fruit had similar SSC to the control fruit, while the SSC of all other biofilm applications fruit was significantly higher than the control. With the exception of U3, all biofilm applications exhibited significantly higher SSC values one week after harvest than those measured at the commercial harvest (Table 5).

**Table 5:** Effect of biofilm application on soluble solids content (SSC), titratable acidity (TA), and vitamin C of ‘0900 Ziraat’ sweet cherry cultivar

Application	Biochemical characteristics							
	SSC (%)			TA (g malic acid 100 mL^–1^)			Vitamin C (mg 100 g^–1^)	
	Harvest	Harvest + 7days		Harvest	Harvest + 7days		Harvest	Harvest + 7days
U1	14.57 c-B	15.20 b-A		0.96 b-A	0.93 a-A		8.25 d-B	10.70 b-A
U2	17.03 a-B	18.50 a-A		1.01 a-A	0.95 a-B		12.65 a-B	14.95 a-A
U3	14.57 c-A	14.77 b-A		1.00 a-A	0.94 a-B		8.90 d-B	10.90 b-A
U4	15.63 b-B	18.57 a-A		1.03 a-A	0.92 a-B		9.30 c-B	10.85 b-A
U5	16.83 a-B	19.10 a-A		0.95 b-A	0.94 a-A		10.60 b-B	14.40 a-A
U6	15.50 b-B	18.67 a-A		0.95 b-A	0.85 b-B		10.50 b-B	15.40 a-A
U7	16.77 a-B	18.13 a-A		0.94 b-A	0.84 b-B		10.55 b-B	14.65 a-A
U8	17.43 a-B	18.80 a-A		0.88 c-A	0.85 b-A		9.35 c-B	15.15 a-A

Means in columns with the same lowercase letters do not significantly differ according to Tukey’s test at *P* < 0.05. Means in rows with the same uppercase letters do not significantly differ according to Tukey’s test at *P* < 0.05

The significant differences in TA content occurred between biofilm applications. At the commercial harvest, the U2, U3, and U4 applications exhibited similar TA contents, which were significantly higher than those observed in the control. In contrast, the U8 application exhibited significantly lower TA levels than the control. One week later, the TA levels of the U6, U7, and U8 applications were found to be similar TA levels, but significantly lower than the control. The majority of applications had significantly higher TA values at the commercial harvest than one week later, except for U1, U5, and U8, which did not significantly differ (Table 5).

A significant difference was observed in the vitamin C content between the various biofilm applications. The biofilm applications demonstrated a significant increase in vitamin C content compared to the control, except for the U3 application at the commercial harvest. At the commercial harvest, the U2 application exhibited a significantly higher vitamin C content (12.65 mg 100 g^–1^) than the other biofilm applications. One week later, the U3 and U4 applications had similar vitamin C contents to the control, whereas the other biofilm applications had significantly higher vitamin C contents than the control (Table 5).

### Total phenolics, total flavonoids and total anthocyanin content

Significant variations in the bioactive compounds were observed among the biofilm applications. The U4 and U5 applications had similar total phenolics contents at the commercial harvest, significantly lower than the control and other biofilm applications. All biofilm applications had significantly higher total phenolics contents one week after harvest than at commercial harvest (Table 6).

**Table 6:** Effect of biofilm application on total phenolics (TP), total flavonoid (TF), and total monomeric anthocyanin (TMA) contents of ‘0900 Ziraat’ sweet cherry cultivar

Application	Bioactive compounds							
	TP (µg GAE g^–1^)			TF (µg QE g^–1^)			TMA (µg cyn-3-gluc g^–1^)	
	Harvest	Harvest + 7days		Harvest	Harvest + 7days		Harvest	Harvest + 7days
U1	232 a-B	348 a-A		111 b-B	197 a-A		6.43 b-B	11.23 c-A
U2	250 a-B	290 b-A		136 a-B	174 b-A		6.65 b-B	10.80 c-A
U3	254 a-B	366 a-A		116 b-B	214 a-A		7.12 a-B	13.25 b-A
U4	185 b-B	272 b-A		104 b-B	173 b-A		5.74 c-B	10.59 c-A
U5	174 b-B	299 b-A		101 b-B	166 b-A		5.50 c-B	10.89 c-A
U6	250 a-B	348 a-A		147 a-B	209 a-A		6.55 b-B	13.57 b-A
U7	246 a-B	359 a-A		134 a-B	203 a-A		6.67 b-B	14.63 a-A
U8	264 a-B	362 a-A		149 a-B	206 a-A		7.25 a-B	14.89 a-A

Means in columns with the same lowercase letters do not significantly differ according to Tukey’s test at *P* < 0.05. Means in rows with the same uppercase letters do not significantly differ according to Tukey’s test at *P* < 0.05

At the commercial harvest, the U2, U6, U7, and U8 applications had similar total flavonoid contents, which were significantly higher than the control and other biofilm applications. One week later, the U2, U4, and U5 applications had similar total flavonoid contents, which were significantly lower than those observed in the control and other biofilm applications (Table 6).

A significant difference was observed in the total monomeric anthocyanin content between biofilm applications at harvest and harvest + 7 days. At the commercial harvest, the total monomeric anthocyanin content of the U3 and U8 applications was found to be similar, although significantly higher than that of the control. In contrast, the U4 and U5 applications exhibited a similar but significantly lower anthocyanin content than the control, while the other biofilm applications demonstrated a similar anthocyanin content to the control. One week after the commercial harvest, the U3, U6, U7, and U8 applications exhibited significantly higher total monomeric anthocyanin than the control, while the U7 and U8 applications exhibited similar total monomeric anthocyanin but significantly higher than the U3 and U6 applications (Table 6).

### Total antioxidant activity

The DPPH assay revealed that the U2, U3, and U5 applications had similar antioxidant activities at commercial harvest, significantly higher than the control and other biofilm applications. One week later, the highest antioxidant activity was observed in the U4 and U5 applications. In addition, it was determined that the antioxidant activity of the U2 and U3 applications was at a similar level but higher than other biofilm applications (Table 7).

**Table 7:** Effect of biofilm application on antioxidant activity (DPPH and FRAP) of ‘0900 Ziraat’ sweet cherry cultivar

Application	Antioxidant activity (µmol TE g^–1^)				
	DPPH			FRAP	
	Harvest	Harvest + 7days		Harvest	Harvest + 7days
U1	0.92 b-B	1.07 c-A		3.48 b-B	6.64 b-A
U2	1.06 a-B	1.71 b-A		4.44 a-B	6.42 b-A
U3	1.12 a-B	1.83 b-A		3.59 b-B	6.92 a-A
U4	0.90 b-B	2.26 a-A		3.36 b-B	5.16 c-A
U5	1.09 a-B	2.19 a-A		3.39 b-B	5.32 c-A
U6	0.89 b-B	1.00 c-A		4.58 a-B	6.55 b-A
U7	0.91 b-B	1.13 c-A		4.68 a-B	6.99 a-A
U8	0.95 b-B	1.09 c-A		4.63 a-B	7.15 a-A

Means in columns with the same lowercase letters do not significantly differ according to Tukey’s test at *P* < 0.05. Means in rows with the same uppercase letters do not significantly differ according to Tukey’s test at *P* < 0.05

At the commercial harvest, the U2, U6, U7 and U8 applications had similar FRAP values, which were significantly higher than those observed in the control. One week later, the U3, U7, and U8 applications exhibited similar antioxidant activities, which were significantly higher than the control and other biofilm applications. All biofilm applications exhibited significantly higher antioxidant activities one week after harvest than at the commercial harvest (Table 7).

## Discussion

Fruit cracking, which is attributed to a number of factors including environmental, morphological, physiological, and genetic [6], is a significant concern in sweet cherry cultivation, reaching rates as high as 90% in some years. The detrimental effects of fruit cracking impact the fruit’s marketability due to injury, unappealing appearance, and increased susceptibility to pathogens [1]. In the study, applying biofilm to ‘0900 Ziraat’ cherry cultivar before harvest decreased fruit cracking rates because it formed a protective film on the fruit, decreasing water intake, enhancing skin elasticity, and possibly increasing calcium content. In particular, the efficacy of biofilm applications was more pronounced in U4 and U6 applications. In both measurement periods, the lowest incidence of cracking was observed in these applications. The fruit’s calcium levels affect the peel’s mechanical properties, impacting fruit cracking [32]. Previous studies have indicated that calcium strengthens cell walls, thereby reducing the fruit’s susceptibility to cracking [24], with more effective results when applied directly to the fruit several times [33]. Fruit cracking is primarily caused by turgor pressure, which arises from rainwater absorbed directly by the fruit peel and cuticle, in addition to water intake through the tree’s transmission system [7]. The primary objective of using biofilm applications, which have been designed with the influence of calcium on fruit cracking in mind, is to impede water absorption through the fruit peel. This is achieved by establishing an artificial coating on the fruit surface, enhancing its integrity, or changing the osmotic potential of the fruit’s surface [13, 28].

The application of transpiration inhibitors, such as Parka, has been demonstrated to limit gas exchange and can therefore adversely affect the chemical composition of the fruit [34]. Our findings indicate that biofilm application results in a reduction in fruit respiration rate, particularly in fruit that have received their initial application. This suggests that there is a limitation in the fruit’s ability to exchange gases. Similarly, Ozturk et al. [16] reported a reduction in respiration rates in fruit that had been treated with biofilm.

In modern sweet cherry cultivation, obtaining larger fruit is a key objective to enhance the economic value. Consumers prefer large fruit due to their visual appeal, taste, and high fruit pulp ratio [28]. This study revealed significant differences in fruit weight occurred between application regimes. Furthermore, the effect of biofilm application on fruit size was found to be inconsistent. Some biofilm applications resulted in larger fruit than the control, whereas the majority produced smaller fruit. Consequently, biofilm application may adversely affect fruit size, potentially due to influence of coating materials on osmotic potential. Previous research has demonstrated that biofilm application does not affect fruit size in sweet cherry [26].

Fruit color is a crucial factor influencing consumer preferences for sweet cherry and is subject to changes during the maturation process. Thus, the primary cultivation goals are to achieve the optimal fruit color specific to the species and to preserve this color postharvest. As the fruit ripens, color changes occur due to increases in bioactive compounds [35]. Edible coatings can reduce color changes by limiting the increase of anthocyanins, which are pivotal in fruit coloration [35]. These coatings affect fruit coloration by altering surface properties and impeding ripening [36]. Nevertheless, Hoagland and Parris [37] reported that such applications restrict changes in anthocyanin and phenolic compound contents, preventing discoloration during cold storage. This effect is attributed to decreased fruit gas exchange, evaporation, and respiration rates and reduced coloration-related enzyme activities in fruit peel [38]. In accordance with these observations, a reduction in fruit coloration was noted in the biofilm-treated fruit, as evidenced by diminished brightness (L*) and red color (a*). Aglar et al. [39] also reported reduced fruit color values at harvest with biofilm application. Nevertheless, Ozturk et al. [16] asserted that pre-harvest biofilm application had no effect on the coloration of jujube.

In fruits such as sweet cherry, with delicate structures, fruit firmness is an important quality trait that determines the storage potential [40]. The softening of fruit occurs as a consequence of the breakdown of cell wall components including pectin substances, hemicellulose, and cellulose break down as maturity progresses [25]. It is of paramount importance to maintain the firmness of fruit flesh for the purpose of marketing. Calcium plays a pivotal role in reinforcing cell wall integrity [24]. The applications of coatings has the effect of reducing the osmotic potential of the plant [28], thereby delaying the ripening of the fruit and maintaining its firmness by providing shell integrity [34]. The biofilm application forms a biofilm coating on the fruit surface and contains calcium, resulting in notable differences in fruit flesh firmness. The biofilm-sprayed sweet cherry fruit had significantly greater firmness than the control fruit in both measurement periods, with slightly reduced firmness one week after harvest compared to commercial harvest. Similarly, Aglar et al. [39] reported higher flesh firmness with biofilm application, with slight losses during cold storage. Ozturk et al. [16] reported that biofilm application did not affect jujube fruit flesh firmness.

The SSC and TA are significant factors in determining the quality of fruit and the optimal time for harvesting. As fruits mature, the hydrolysis of undissolved polysaccharides in simple sugars increases SSC and decreases TA [41], as observed in our study. Transpiration inhibitors such as Parka can result in adverse affects on the chemical composition of fruit by limiting gas exchange [34]. In contrast to the findings of this study, Measham et al. [26] reported a reduction in SSC following the application of biofilm in sweet cherry in the United State, Australia, and Türkiye. Similarly, Aglar et al. [39] reported a reduction in SSC rates in fruit treated with a biofilm, but no significant differences in TA rates. Ozturk et al. [16] observed that biofilm application did not affect SSC and TA in jujube fruit.

Sweet cherry contain bioactive compounds with antioxidant properties, including polyphenols, vitamins, anthocyanins, and carotenoids. The concentration of these compounds varies depending on the maturity of the fruit [42]. In general, as fruit matures, the concentration of these compounds increases [35]. The use of edible coatings, such as Parka and *Aloe vera* gel can serve to limit the increase in these bioactive compounds during the maturation of the fruit [39, 43]. However, we observed inconsistencies in the effect of biofilm application on total phenolic, total flavonoid, total monomeric anthocyanin, and antioxidant activity. Some applications demonstrated no effect, while others exhibited positive effects, and yet others exhibited negative impacts. Aglar et al. [39] reported lower levels of vitamin C, total phenolic, total flavonoid, total monomeric anthocyanin, and antioxidant activity in biofilm -applied sweet cherry. Furthermore, they observed a reduction in vitamin C content as the fruit ripened postharvest, while other bioactive compounds exhibited an increase, particularly in the control fruit. Nevertheless, Ozturk et al. [16] proposed that biofilm-treated jujube exhibited higher concentrations of bioactive compounds and antioxidant activity.

## Conclusions

Parka (biofilm) application was found to be reduce fruit cracking, reduce fruit size, delay coloration, increase flesh firmness and vitamin C content in sweet cherry fruit. Nevertheless, the impact of the application on total phenolic, flavonoid, and monomeric anthocyanin contents and antioxidant activity was inconsistent. The study was determined that cracking, a major concern for the sweet cherry industry and consumers worldwide, could be reduced by biofilm applications, and that postharvest life could be extended by increasing the fruit firmness of sweet cherries. It was concluded that biofilm could be used as a sustainable tool for growers to increase the profitability and marketability of sweet cherries.

## Data Availability

Data will be available on request to corresponding authors.

## Abbreviations

CIE: Commission de International de I’Eclairage
DPPH: 2,2-Diphenyl-1-picrylhydrazyl
FRAP: Ferric Reducing Antioxidant Power
GAE: Gallic acid equivalent
h: Hour
min: Minute
µg: Microgram
pH: Power of hydrogen
s: Second
SSC: Soluble solids content
TA: Titratable acidity
TCA: Trichloroacetic acid
TE: Trolox equivalent
TMA: Total monomeric anthocyanin
QE: Quercetin equivalent
